# Potential favorable action of sodium-glucose cotransporter-2 inhibitors on sudden cardiac death: a brief overview

**DOI:** 10.3389/fcvm.2023.1159953

**Published:** 2023-05-12

**Authors:** Tatsuya Sato, Hidemichi Kouzu, Toshiyuki Yano, Ichiro Sakuma, Masato Furuhashi, Noritsugu Tohse

**Affiliations:** ^1^ Department of Cellular Physiology and Signal Transduction, Sapporo Medical University School of Medicine, Sapporo, Japan; ^2^ Department of Cardiovascular, Renal, and Metabolic Medicine, Sapporo Medical University School of Medicine, Sapporo, Japan; ^3^ Caress Sapporo Hokko Memorial Clinic, Sapporo, Japan

**Keywords:** SGLT2 inhibitors, sudden cardiac death (SCD), ventricular arrhythmia (VAs), electrophysiology, mini review

## Abstract

The primary pharmacological action of sodium-glucose co-transporter 2 (SGLT2) inhibitors is to inhibit the reabsorption of glucose and sodium ions from the proximal tubules of the kidney and to promote urinary glucose excretion. Notably, several clinical trials have recently demonstrated potent protective effects of SGLT2 inhibitors in patients with heart failure (HF) or chronic kidney disease (CKD), regardless of the presence or absence of diabetes. However, the impact of SGLT2 inhibitors on sudden cardiac death (SCD) or fatal ventricular arrhythmias (VAs), the pathophysiology of which is partly similar to that of HF and CKD, remains undetermined. The cardiorenal protective effects of SGLT2 inhibitors have been reported to include hemodynamic improvement, reverse remodeling of the failing heart, amelioration of sympathetic hyperactivity, correction of anemia and impaired iron metabolism, antioxidative effects, correction of serum electrolyte abnormalities, and antifibrotic effects, which may lead to prevent SCD and/or VAs. Recently, as possible direct cardiac effects of SGLT2 inhibitors, not only inhibition of Na^+^/H^+^ exchanger (NHE) activity, but also suppression of late Na^+^ current have been focused on. In addition to the indirect cardioprotective mechanisms of SGLT2 inhibitors, suppression of aberrantly increased late Na^+^ current may contribute to preventing SCD and/or VAs via restoration of the prolonged repolarization phase in the failing heart. This review summarizes the results of previous clinical trials of SGLT2 inhibitors for prevention of SCD, their impact on the indices of electrocardiogram, and the possible molecular mechanisms of their anti-arrhythmic effects.

## GRAPHICAL ABSTRACT

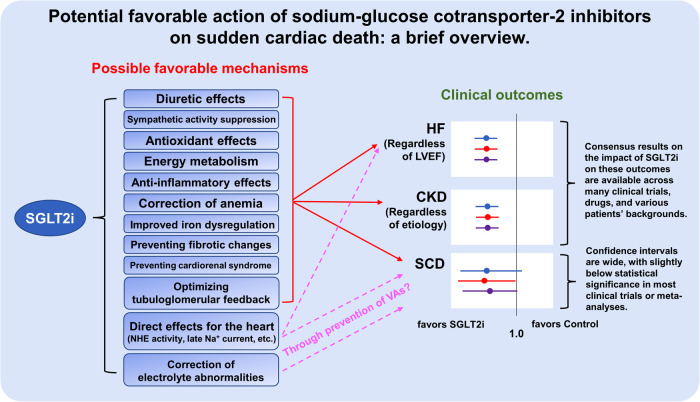

## Introduction

Sodium-glucose co-transporter 2 (SGLT2) inhibitors are increasingly gaining attention for their cardiorenal protective effects and their clinical significance (1). However, compared to the robust favorable effects of SGLT2 inhibitors on diabetes mellitus, heart failure, and chronic kidney disease, results of studies on their clinical impact on sudden cardiac death (SCD) have been inconsistent. Therefore, it is worth evaluating whether SGLT2 inhibitors can prevent the incidence of SCD in the presence or absence of diabetes mellitus, heart failure, or chronic kidney disease. This mini-review focuses on the results of recent clinical trials of SGLT2 inhibitors for prevention of SCD, the effects of SGLT2 inhibitors on ventricular indices of an electrocardiogram, and the possible molecular mechanisms of the anti-arrhythmic effects of SGLT2 inhibitors.

## General background on SGLT2 inhibitors and their potential impact on SCD

A major pharmacological action of SGLT2 inhibitors is to inhibit the reabsorption of glucose and sodium ions and to promote urinary glucose excretion by inhibiting SGLT2, which is abundant at the brush border membrane in the early S1 segment of the proximal tubules of the kidney (2). Thus, SGLT2 inhibitors were initially developed and introduced to the market as oral hypoglycemic agents with insulin-independent hypoglycemic effects. However, a number of large clinical trials in recent years have demonstrated the potent cardiorenal protective effects of SGLT2 inhibitors, and interestingly, their protective effects have been observed in both patients with and those without diabetes mellitus (3–8). Several SGLT2 inhibitors are now also recommended for the treatment of chronic heart failure or chronic kidney disease as well as diabetes mellitus (9–11). Compared to SGLT1, which has been reported to be expressed in multiple organs and cells (12), SGLT2 is expressed almost exclusively in the proximal tubules (13), and it is reasonable to assume that the cardiorenal protective effect of SGLT2 inhibitors is exerted via the pathway through the proximal tubule in the kidney. The cardiorenal protective mechanisms of SGLT2 inhibitors are thought to be complex, including diuretic effects that less activate the renin-angiotensin-aldosterone system with little change in intravascular volume (14, 15), suppression of sympathetic hyperactivity (16), antioxidant effects (17, 18), correction of impaired energy metabolism (19, 20), anti-inflammatory effects (21), improvement of anemia via increased erythropoietin production (22), improved iron metabolism (23), preventing fibrotic changes (24, 25), and preventing effects for the development of cardiorenal syndromes (26). Nevertheless, direct effects of SGLT2 inhibitors on cardiomyocytes, including inhibition of Na^+^/H^+^ exchanger (NHE) activity (27, 28) and suppression of late Na^+^ current (29–31), have also recently been reported, although it has been reported that SGLT2 inhibitors do not have an inhibitory effect on NHE activity in intact cardiomyocytes (32), and the precise mechanisms how SGLT2 inhibitors directly bind to cardiomyocytes that lack SGLT2 remain undetermined.

**Figure 1:**
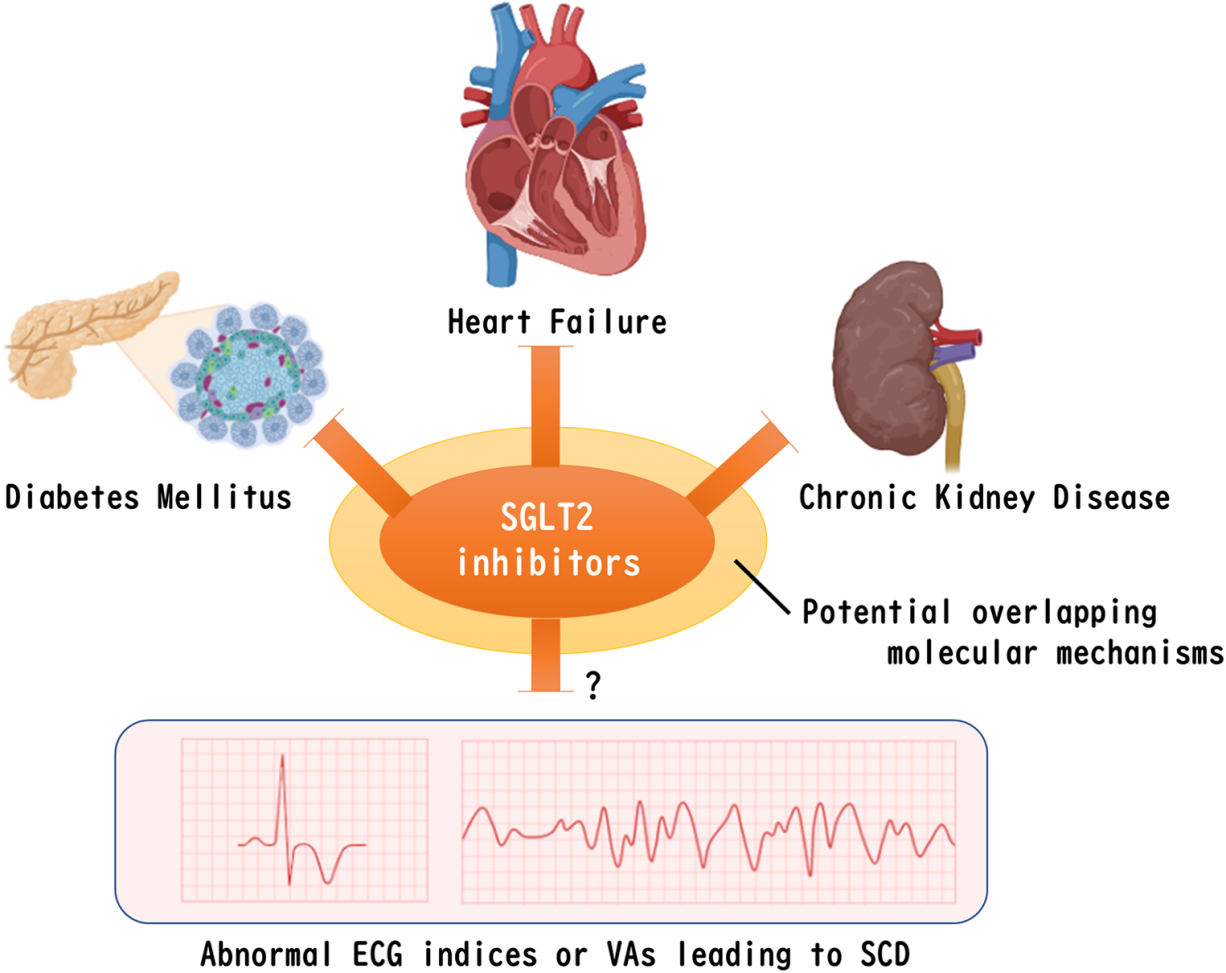
Remaining question of whether SGLT2 inhibitors improve abnormal ventricular ECG indices or VAs that leads to SCD. Despite the possible existence of a common molecular mechanism for SGLT2 inhibitors to ameliorate diabetes, heart failure, and chronic kidney disease, clinical evidence for a positive effect on VAs that leads to SCD has not yet been determined. The figure was created by using licensed BioRender. SGLT2, sodium-glucose co-transporter 2, ECG, electrocardiogram, VAs, fatal ventricular arrhythmias, SCD, sudden cardiac death.

Although the absolute event rate of SCD is not as high as that of heart failure or chronic kidney disease (33), the impact of SCD is devasting because the people left behind have to deal with the sudden loss of a dear one without any preparation caused by a SCD event. It has been noted that fatal ventricular arrhythmias (VAs), regardless of the presence or absence of ischemic heart disease or heart failure, are involved in the majority of causes of SCD, although age and gender differences in the causes of SCD have been reported (34, 35). In addition, it has been known that the incidence of SCD via VAs is higher in patients with pre-existing heart diseases, including ischemic heart disease and cardiomyopathy (36). Indeed, an implantable cardioverter defibrillator (ICD) is recommended for the prevention of SCD in patients with documented VAs and in patients with heart disease (37). Even in individuals without cardiac disease, a recent genome-wide association study (GWAS) has proposed that risk factors for cardiac disease such as diabetes, obesity, hypertension, and dyslipidemia are risks for SCD in addition to QT interval prolongation (38), which is a well-established index of an electrocardiogram (ECG) for a risk of SCD. Interestingly, many of the pathophysiological mechanisms involved in the cardioprotective effects of SGLT2 inhibitors appear to overlap with those involved in the pathogenesis of SCD (39). However, clinical evidence on the favorable effects of SGLT2 inhibitors in preventing SCD or inhibiting VAs has been limited and remains controversial (Figure 1).

## Clinical studies of SGLT2 inhibitors focusing on SCD

Investigations to determine whether SGLT2 inhibitors reduce the incidence of SCD were examined in the EMPA-REG OUTCOME trial, which demonstrated for the first time the potential of SGLT2 inhibitors to reduce cardiovascular events in patients with type 2 diabetes mellitus who have cardiovascular disease (40). In that trial, SCD was clearly defined as the death that occurred unexpectedly in a previously stable patient and included the following conditions: (1) witnessed and instantaneous without new or worsening symptoms, (2) witnessed within 60 min of the onset of new or worsening cardiac symptoms, (3) witnessed and attributed to an identified arrhythmia, (4) individuals unsuccessfully resuscitated from cardiac arrest or successfully resuscitated from cardiac arrest but who died within 24 h without identification of a non-cardiac etiology, and (5) unwitnessed death with no conclusive evidence of another non-cardiovascular death. In a sub-analysis of this study, the pooled empagliflozin group had a 31% lower risk of SCD compared to the placebo group, but did not reach statistical superiority (event rate: 1.1% in the empagliflozin group vs. 1.6% in the placebo group; hazard ratio: 0.69; 95% confidence interval: 0.45–1.04) (41). In a similar study with other SGLT2 inhibitors, the DECLARE-TIMI 58 trial (42), treatment with dapagliflozin significantly reduced by 17% the primary endpoint of heart failure plus cardiovascular death in patients with type 2 diabetes mellitus who had high risks for cardiovascular events, including those with existing cardiovascular diseases, although such cardioprotective effects were limited to the onset of heart failure. Interestingly, the results of the DECLARE-TIMI 58 trial revealed that SCD accounted for 58% of all deaths, raising the concern that SCD in this study may include deaths from multiple causes that are considered unassessable (43). Thus, that study also highlights the difficulty of etiological interpretation using SCD as an endpoint in such large randomized controlled clinical trials.

Nevertheless, a meta-analysis of multiple clinical trials may be useful for evaluating whether SGLT2 inhibitors reduce the incidence of SCD, which is not expected to be as common an event as the development of heart failure or chronic kidney disease. Interestingly, however, the results of two recent meta-analyses were different. Fernandes et al. performed a meta-analysis of a total of 34 randomized control trials including 63,166 patients with type 2 diabetes mellitus or heart failure for evaluating the effect of SGLT2 inhibitors on SCD (44). They showed that treatment with SGLT2 inhibitors was associated with a significant reduction in the risk of SCD (odds ratio: 0.72; 95% confidence interval: 0.54–0.97) compared with that in the control group. On the other hand, Sfairopoulos et al. conducted a meta-analysis of a total 19 randomized control trials that enrolled 55,590 patients with type 2 diabetes mellitus, heart failure, or chronic kidney disease for evaluating the effect of SGLT2 inhibitors on SCD (45). They concluded that there was no significant association between therapy with SGLT2 inhibitors and SCD (risk ratio: 0.74; 95% confidence interval: 0.50–1.08) and that treatment with SGLT inhibitors was not associated with a lower risk of VAs (risk ratio: 0.84; 95% confidence interval: 0.66–1.06). In a recent cohort study other than those included in the meta-analysis, Eroglu et al. analyzed data for 152,591 patients with type 2 diabetes mellitus to examine whether the use of SGLT2 inhibitors is more closely associated than the use of other anti-diabetic agents with SCD (46). They showed that the use of SGLT2 inhibitors was associated with a trend of lower event rates for SCD compared to other antidiabetic drugs after adjustment for common risk factors of SCD, but that this association did not reach statistical significance (hazard ratio: 0.62; 95% confidence interval: 0.38–1.01), while the hazard ratio for all-cause mortality for the use of SGLT2 inhibitors vs. other anti-diabetic drugs was significantly low (hazard ratio: 0.43; 95% confidence interval: 0.39–0.48). Of note, these different results may be related not only to the kind of patients, but also to a lower event rate of SCD because there were large confidence intervals for SCD events among those studies.

On the other hand, regarding the preventive effect of SGLT2 inhibitors on SCD in a heart failure population with expected higher SCD event rates, a recent combined analysis of DAPA-HF trial [in which the left ventricular ejection fraction (LVEF) of the participants was ≤40%] and DELIVER trial (LVEF >40%) demonstrated that treatment with the SGLT2 inhibitor dapagliflozin resulted in lower rates of SCD in patients with heart failure independent of the degree of LVEF (hazard ratio: 0.84; 95% confidence interval: 0.70–1.01), although the effect marginally failed to reach the nominal threshold for statistical significance (47). Indeed, a very recent meta-analysis exclusively for patients with heart failure, including 11 randomized control trials (10,796 patients received SGLT2 inhibitors and 10,796 patients received placebo), demonstrated that treatment with SGLT2 inhibitor was associated with a significant reduction in the risk of SCD (risk ratio: 0.68; 95% confidence interval: 0.48–0.95) (48). It has also been reported that patients with both type 2 diabetes mellitus and post-myocardial infarction have a higher incidence of SCD, even in the absence of residual ischemia (49). Interestingly, a recent observational study reported that patients with type 2 diabetes mellitus who were using SGLT2 inhibitors prior to the onset of myocardial infarction had a significantly lower rate of in-hospital VAs after myocardial infarction than those who were not using SGLT2 inhibitors (50). Thus, the preventive effect of SGLT2 inhibitors on SCD, if any, may be more prominent in high-risk populations such as patients with heart failure, ischemic heart disease, and cardiomyopathy.

Overall, it appears that SGLT2 inhibitors at least tend to decrease, rather than increase, events of SCD, but further *de novo* studies that take into account factors of a small event rate of SCD compared with that of heart failure or kidney disease, clear definition of SCD, and patients' backgrounds, are needed to conclude whether SGLT2 inhibitors can prevent SCD and/or VAs.

## Effect of SGLT2 inhibitors on ventricular indices of electrocardiograms and underlying mechanisms for their potential favorable action in the heart

To assess the safety of a newly developed drug, it must be confirmed that the drug does not affect ECG indices, especially the QT interval. Consistent with this criterion, it has been reported that SGLT2 inhibitors do not affect ECG indices, including the QT interval, in healthy individuals (51, 52). On the other hand, patients with cardiomyopathy or heart failure, regardless of their etiology, often represent an abnormal ECG that reflects impaired excitation-contraction coupling and disturbance of conduction. Interestingly, there have even been attempts to detect certain cardiac diseases through ECG diagnosis using artificial intelligence in recent years (53). QT dispersion, a classic index of ventricular repolarization heterogeneity, has been reported to be increased in individuals with cardiomyopathy, ischemic heart disease, prolonged QT syndrome, and left ventricular hypertrophy of various etiologies (54), and an increase in its index is associated with overall mortality (55). Furthermore, Tpeak-Tend, defined as the interval from the peak of the T wave to the end of the T wave in an ECG at a chest lead, has also been reported to reflect ventricular repolarization heterogeneity and to be useful for predicting VAs and SCD (56, 57). In patients with type 2 diabetes mellitus, prolonged QT dispersion or Tpeak-Tend interval have been reported by the authors and other groups to be improved by SGLT2 inhibitors (58–60). It should be noted that there is a report showing that SGLT2 inhibitors had no effects on ECG indices including QT interval in patients with type 2 diabetes in a cohort study, although indices of ventricle repolarization heterogeneity were not assessed in that study (61). Since conventional hypoglycemic agents have no effect on these indices of ventricular repolarization heterogeneity (62), the improvement of ventricular repolarization heterogeneity must be independent of its glycemic control. Indeed, the degree of improvement in ventricular repolarization heterogeneity was shown to be associated with the degree of reduction of elevated blood pressure (58) or weight loss (59). Furthermore, an abnormal QRS-T angle, another index of ventricular repolarization heterogeneity and a predictor for SCD (63), has been reported to be improved by SGLT2 inhibitors in patients with type 2 diabetes mellitus with known cardiovascular diseases (64). Taken together, the results of studies indicate that SGLT2 inhibitors may improve impaired indices of ventricular repolarization heterogeneity, but whether similar results are observed in patients with heart failure or chronic kidney disease in the absence of diabetes mellitus requires further investigation.

In case that SGLT2 inhibitors do in fact ameliorate the abnormal heterogeneity of ventricular repolarization, its underlying molecular mechanisms are of interest. As a novel molecular mechanism of SGLT2 inhibitors on isolated cardiomyocytes, several studies using rodent animals have shown that SGLT2 inhibitors suppress late Na^+^ current, which is increased by oxidative stress, in a calmodulin-dependent protein kinase II (CAMKII)-dependent manner (30, 31). Late Na^+^ current can contribute to prolonged action potential duration by increasing an inward current during the repolarization phase (65). If the finding is also observed in the human myocardium, modification of increased late Na^+^ current by SGLT2 inhibitors may be a mechanism for improving abnormally prolonged ventricular repolarization, resulting in amelioration of impaired ECG indices of ventricular repolarization heterogeneity. In addition to the direct effects of SGLT2 inhibitors, it has been reported that systemic administration of SGLT2 inhibitors exerts antioxidant effects via increased ketone bodies in the blood (66) and improved metabolic flexibility with restored mitochondrial function in the heart (67). These indirect effects of SGLT2 inhibitors may also be involved in the suppression of cardiac late Na^+^ current via reduced oxidative stress. Another possibility of impaired repolarization in the failing cardiomyocytes is that voltage-gated K^+^ currents, which contribute to regulating repolarization heterogeneity (68), have been reported to be reduced in rodent models of diabetes mellitus and/or heart failure (69, 70). Indeed, there are reports that SGLT2 inhibitors can promote K^+^ currents that enhance repolarization in different species (71–74). Besides acting on ion channels themselves, SGLT2 inhibitors may also improve ECG abnormalities via improved serum electrolytes. In fact, SGLT2 inhibitors have been suggested to improve abnormal serum levels of potassium and/or magnesium (75–77), leading to improved ECG abnormalities independently of the direct effect of ion channels. In addition, increased myocardial stretch is known to promote arrhythmogenesis via modification of calcium handling (78), activation of stretch-induced angiotensin II type 1 receptor signaling (79), and activation of stretch-induced ion currents (80). Since it has been reported that SGLT2 inhibitors have a potential to reduce cardiac volumes (81), improved myocardial stretch by SGLT2 inhibitors in patients with heart failure, at least partially, could explain the anti-arrhythmic effect of SGLT2 inhibitors. Furthermore, the arrhythmogenic potential is also promoted not only by single myocyte abnormalities, but also by aberrant conduction via myocardial injury-induced scar (82). Indeed, a recent study demonstrated that administration of SGLT2 inhibitors ameliorates ischemia/reperfusion injury via reduction of microvascular obstruction and increases myocardial salvage (83), suggesting that the cardioprotective effect of SGLT2 inhibitors on myocardial injury may be indirectly related to another benefit of SGLT2 inhibitors on VAs. These reported findings suggest that SGLT2 inhibitors have potential protective effects on SCD and/or VAs via electrophysiologic mechanisms, and further studies in this area are warranted.

## Limitation

The recent COVID-19 pandemic, at least partially, has affected several recent clinical trials, although it has been shown that SGLT2 inhibitors failed to improve outcomes in patients with COVID-19 (84). The possibility that some events of SCD may have been caused by COVID-19 rather than VAs or cardiac events cannot be ruled out. Therefore, caution is needed in interpreting the results of recent clinical trials and the meta-analysis including the studies that were affected by COVID-19. To address questions of whether SGLT2 inhibitors decrease events of SCD, it is necessary to obtain direct evidence of whether SGLT2 inhibitors reduce VAs or not in various patients. For example, results of ongoing clinical trials, including those evaluating the incidence of arrhythmic events before and after SGLT2 inhibitor treatment in patients with type 2 diabetes mellitus with ICD (85) and in patients with heart failure with ICD (86), may provide a major clue for revealing the association between the use of SGLT2 inhibitors and the incidence of SCD as well as VAs. Finally, in terms of arrhythmias, this review did not address the effect of SGLT2 inhibitors on atrial fibrillation, but SGLT2 inhibitors are likely to be effective in preventing the onset of atrial fibrillation (87, 88).

## Conclusion and perspective

SGLT2 inhibitors have the potential to improve abnormal electrophysiological remodeling in the failing myocardium, possibly leading to a reduction in the incidence of SCD and VAs. Clinical studies using SGLT2 inhibitors with a clear definition of SCD and VAs may help to answer the remaining question of whether SGLT2 inhibitors can reduce SCD and/or VAs. Resolving these questions also has the potential to advance current treatment strategies for the prevention of SCD.
